# Preference Signals and Interview Invitations: Insight Into Recent Updates to the Oto‐HNS Residency Application Process

**DOI:** 10.1002/oto2.70024

**Published:** 2024-10-23

**Authors:** Radhika Duggal, Kyra Osborne, Alan Kominsky, William S. Tierney

**Affiliations:** ^1^ Cleveland Clinic Lerner College of Medicine Case Western Reserve University Cleveland Ohio USA; ^2^ Department of Otolaryngology ‐ Head and Neck Surgery Cleveland Clinic Foundation Cleveland Ohio USA

**Keywords:** medical school, otolaryngology, research, residency application, residents

## Abstract

**Objective:**

While students in the 2023 Otolaryngology–Head and Neck Surgery (Oto‐HNS) residency match were allowed 7 preference signals, this number increased to 25 for the 2024 match with the goal of reducing the overall application volume. We sought to understand the impact of this change to application volume and interview patterns.

**Study Design:**

Cross‐sectional survey.

**Setting:**

Program directors of US Oto‐HNS residency programs were invited to participate in an anonymous, electronic survey.

**Methods:**

An anonymous REDCap questionnaire was sent via email to all current Oto‐HNS program directors in January 2024. Data were analyzed using R Version 4.3.1.

**Results:**

Forty‐four program directors completed the survey. While programs received a median [interquartile range] of 400 [363, 445] applications last year, this year they reported receiving 295 [233, 339] applications of which a median of 110 applicants (40%) signaled the program. While the median percent of applicants who were interviewed by a program was 16%, the percent of interviews among applicants who had signaled the program was 37%. Of all interviews, nearly all (median 100% [91, 100]) were of applicants who had signaled the program. Finally, 40 (91%) of program directors reported that signaling played an important role in deciding to interview a candidate.

**Conclusion:**

Preference signals play an important role in a residency program's decision to interview a candidate. Our findings suggest that the implementation of preference signals successfully decreased the average number of applications received by each program and that medical students applying to more programs than available signals may experience diminishing returns.

The Otolaryngology–Head and Neck Surgery (Oto‐HNS) residency match has become increasingly selective over time, with 573 students applying for approximately 382 positions in 2024.^1^ As the number of applicants continues to increase, the number of residency positions has not increased at the same rate. As such, the qualifications of applicants are becoming increasingly competitive, with applicants seeking a greater volume of experiences, such as research, to bolster their application.^2^ Along with rising criteria for applicants, applicants also increased the number of applications submitted in the past 5 years from an average of 64 per applicant in 2019 to 82 per applicant in 2023.^1^ This has resulted in a growing number of applications received by Oto‐HNS programs each year, making holistic review of applications more difficult.^3^

The Oto‐HNS residency match process has undergone numerous changes in the past several years. Recently in 2022, preference signaling was introduced as a mechanism through which Oto‐HNS residency applicants could demonstrate interest in particular residency programs. While students were permitted 5 signals in the 2022 match, they were permitted 7 in the 2023 match. In the 2024 match, the number of preference signals was increased to 25. This system will remain in place for the 2025 match cycle. Preference signaling was implemented with the goals of aligning program and applicant interests during interview selection while mitigating surges in application numbers and allowing for a more comprehensive applicant review.^4^, ^5^, ^6^ In addition to these goals, a previous study of the 2021 to 2022 match cycle demonstrated that interviews were more evenly distributed among applicants after the implementation of preference signaling.^7^ The new “high signal approach” specifically aims to de‐incentivize the rising number of applications submitted by each Oto‐HNS applicant and instead encourage thoughtful consideration of programs to which an application is submitted.^8^ Unsurprisingly, this change in application structure has profound impact on which applicant matches into a given program. In the orthopedic surgery residency match, which allows up to 30 preference signals, it has been demonstrated that the chances of an applicant receiving an interview at a program which was not signaled is low, with 1 study estimating only 0.92% of applicants receiving an interview invitation.^9^, ^10^

While this trend to match “signaling” applicants exists in the orthopedic surgery residency match process, there is currently a paucity of data to confirm the relation between interview invitations and preference signals under the current “high signal approach” in the Oto‐HNS residency application process. Therefore, in this study, we aimed to assess impact of and attitudes toward the new preference signaling model by surveying current Oto‐HNS residency program directors regarding their use of preference signals in the decision to interview a candidate. Specifically, given the paucity of existing objective data, we aimed to characterize the interview yield for students who did and did not signal a program.

## Methods

A list of all current Oto‐HNS residency program directors and their emails was obtained from the Otolaryngology Program Director Organization's website. If an email address was flagged as not valid, the current program director and email was manually identified through the individual residency program's website.

An anonymous REDCap questionnaire was sent via email to all Oto‐HNS program directors. The questionnaire was emailed to all program directors a total of 3 times over a 6‐week period between January and February 2024. Data were summarized using R Version 4.3.1. A Wilcoxon Signed‐Rank test was utilized to test whether the number of applications received by programs differed between the 2023 and 2024 match cycle. Institutional review board review was not required as this research involved fully anonymized, program‐level survey data pertaining to the residency program and was therefore considered “non‐human subject research.”

## Results

A total of 44 (35%) of program directors participated in this survey. To encourage responses, program identifying information was not collected from survey respondents. As demonstrated in Table 1, while programs reported receiving a median [interquartile range, IQR] of 400 [363, 445] applications in the 2023 match cycle, they reported receiving 295 [233, 339] applications in the 2024 match cycle. The median reduction in the number of applications received was 100 [57, 168].

**Table 1:** Number of Applications and During the 2023 and 2024 Oto‐HNS Match

	Median [IQR]	
Number of applications for 2023 match	400 [363, 445]	*P* < .01
Number of applications for 2024 match	295 [233, 339]	
Reduction in number of applications	100 [57, 168]	

Abbreviations: IQR, interquartile range; Oto‐HNS, Otolaryngology–Head and Neck Surgery.

**Figure 1:**
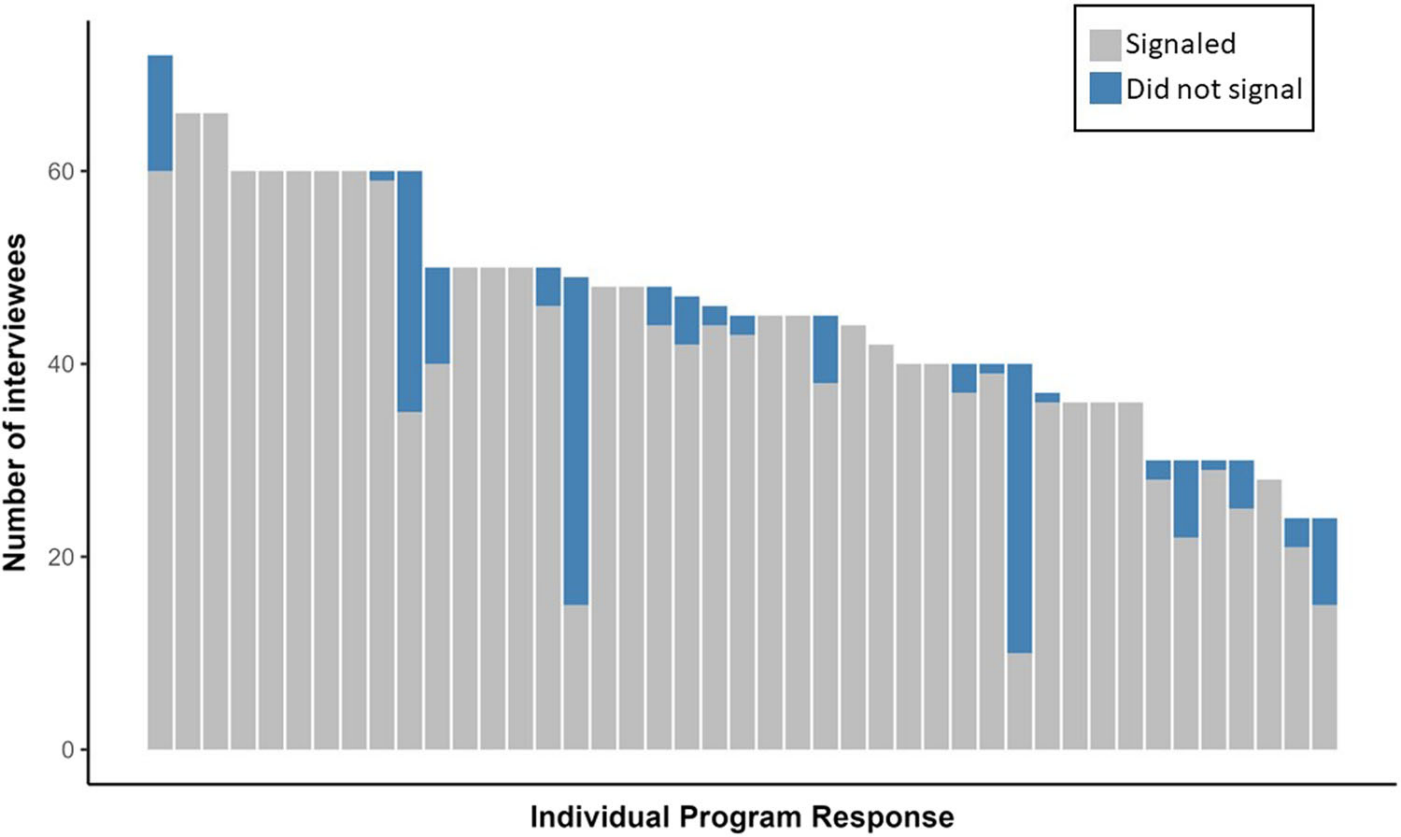
Number of interview invitations sent to individuals who did (gray) and did not (blue) signal the program.

**Figure 2:**
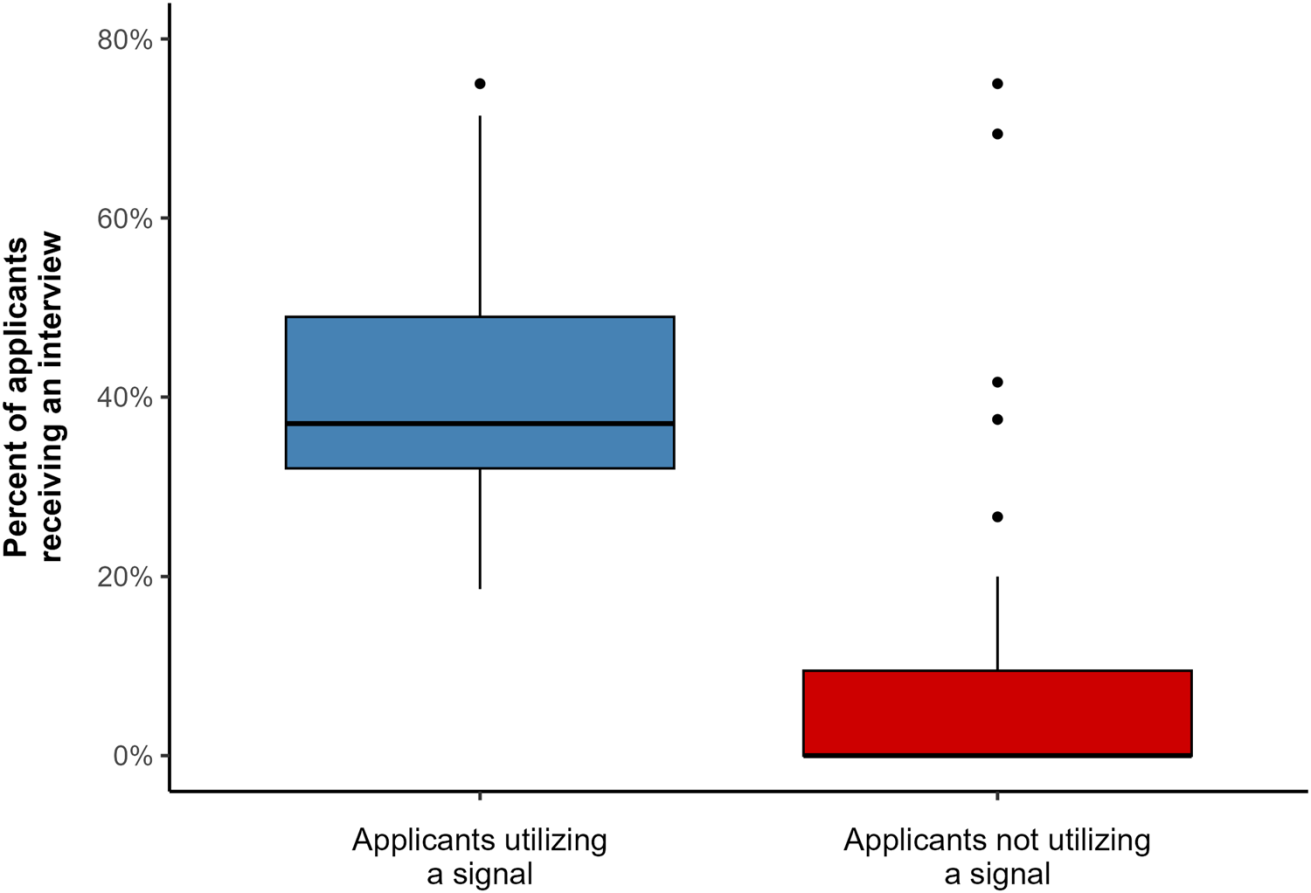
Percent of applicants receiving an interview, stratified by whether the applicant signaled the program.

In the 2024 match cycle, programs reported receiving a median of 110 [80, 131] signals (Table 2 **)**. This corresponds to approximately 40% [31, 50] of all applicants to a program also signaling the program. Of all reported applicants, programs reported a median of 16% [14, 19] were interviewed. Of interviewed applicants, a median of 100% [91, 100] of applicants had signaled the program as depicted in Figure 1. When stratifying the percent of applicants who were interviewed by whether the applicant had signaled the program, we found that a median of 37% [32, 49] of applicants signaling a program were interviewed compared to only 0% [0, 9.5] of applicants who did not signal the program (Figure 2 **)**.

**Table 2:** Number of Signals Received and Applicants Interviewed

	Median [IQR]
Number of signals received	110 [80, 131]
Percent of applicants who signaled the program	40% [31%, 50%]
Total number of applicants interviewed	46 [39, 53]
Percent of all applicants that were interviewed	16% [14%, 19%]
Percent of signaling applicants that were interviewed	37% [32%, 49%]
Percent of applicants that did not signal and were interviewed	0% [0%, 9.5%]
Percent of applicants interviewed that signaled	100% [91%, 100%]

Abbreviation: IQR, interquartile range.

**Figure 3:**
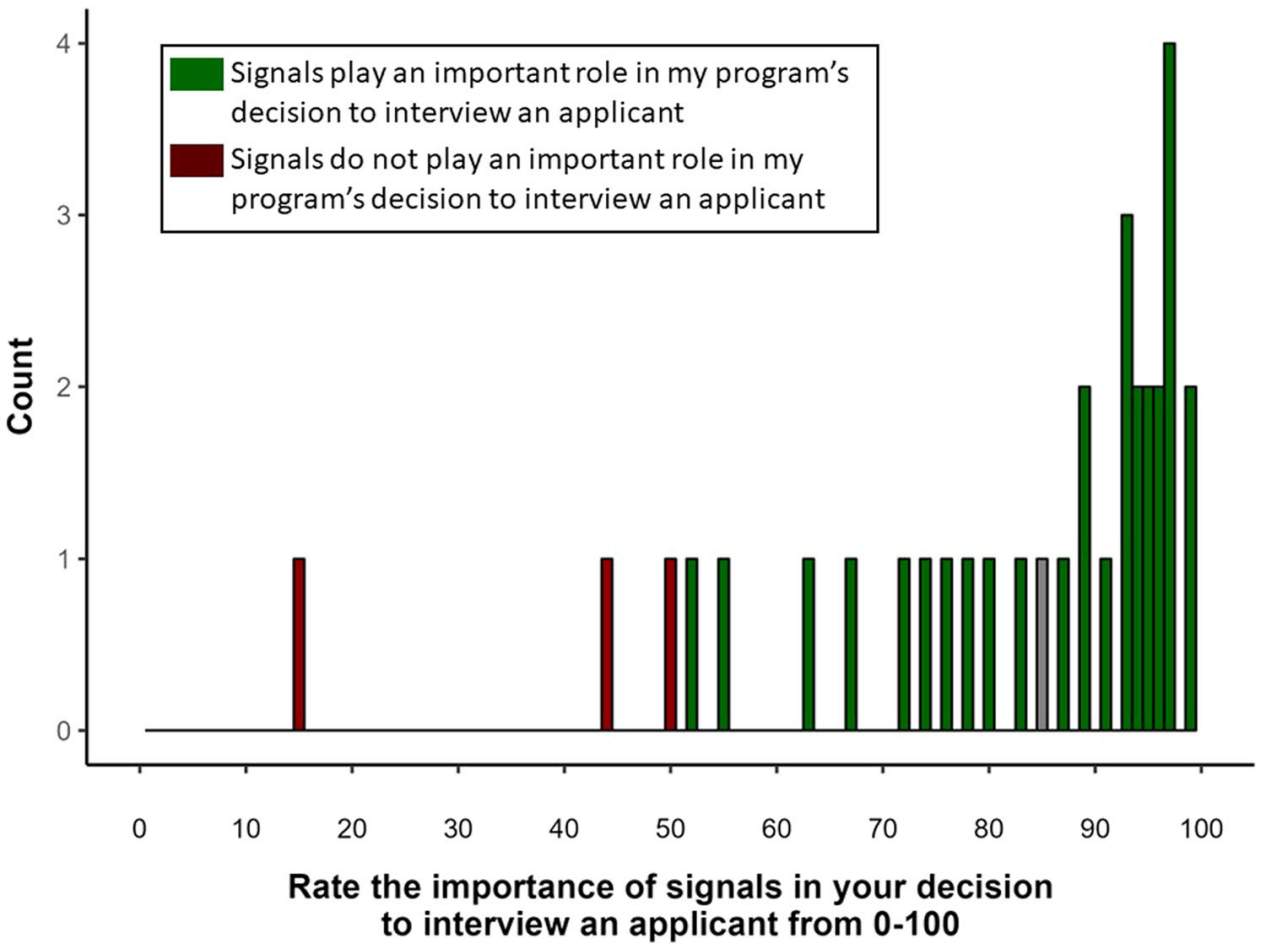
Responses of program directors regarding the role of preference signals in the decision to interview a candidate.

Finally, Figure 3 describe the perceptions reported by program directors toward program signals. We found that 40 (91%) of program directors reported feeling that signals play an important role in deciding to interview a candidate. When program directors were asked to rank the importance of signals on a scale from 1 to 100, the median score was 94 [79, 99].

## Discussion

With recent changes in the residency application process, including the transition of the USMLE Step 1 exam to a pass/fail scoring system, residency program directors must adapt to the new applicant information which is available for consideration.^11^ While away rotations were previously the main avenue through which an applicant could demonstrate interest in a program, preference signaling creates a new mechanism to demonstrate interest. Given the transition to permitting up to 25 preference signals in the 2024 Oto‐HNS match cycle, it is important to understand the impact of preference signals in a residency program's decision to interview a candidate. In this study, we aimed to understand the utilization of preference signals in the interview process.

Compared to the 2023 match cycle, programs reported receiving a median [IQR] of 100 [57, 168] fewer applications in the 2024 match cycle. This drop was expected, as applicants were likely to reduce the number of programs to which they apply in consideration of previous studies demonstrating the impact of high‐volume preference signaling in other specialties. This finding does support the use of signaling to reduce application burden for both applicants and programs. Compared to data in the January 2024 preliminary ERAS report, which reported an average of 354 applications per program in 2023 and 259 in 2024, our study found a modestly greater volume of applications received per program (average of 396 and 283, respectively).^1^ While this discrepancy is likely secondary to response bias introduced by the design of our study, it sheds important light to considerations regarding the bias of our study. That is, based on the average number of applications received, our data is more characteristics of programs which receive a large number of applications each year.

Our results additionally demonstrate that the interview yield of applications submitted to programs that are not signaled is low. We found that each program interviewed a median of 16% [14%, 19%] of all applicants. While signaling increased the percent of interviewed applicants to 37% [32%, 49%], of applicants who did not signal the program a median of 0% [0%, 9.5%] were interviewed. This difference in interview yield is consistent with a previous study of Oto‐HNS applicants which demonstrated that individuals who signal a program have a significantly greater chance of receiving an interview.^12^ However, this previous study found the rates of interview invitations among applicants who signaled the program to be 58% (compared to 14% in applicants who did not signal the program). The difference between these previously reported rates and those found in our study is likely secondary to the differing number of preference signals available at the time of each study. With 5 preference signals available at the time of the previous study, it was clear that students had a limited number of signals, and not all programs a student is potentially interested in could receive a signal. Therefore, it is understandable that in the current paradigm of 25 signals available, the decline in interview rates among candidates who did not provide a preference signal to a program is more exaggerated.

Finally, our study assessed the attitudes of program directors toward preference signaling. Forty programs (91%) responded that signals play an important role in their decision to interview a candidate. When offered a slider from 0 to 100 to quantify the importance of signals in the decision to interview a candidate, the median score was 94 [79, 99]. This is again consistent with previous assessments which demonstrated that 91% of program directors surveyed regarding the signaling process following the 2021 match responded that they strongly favored continuing the signaling process.^12^

Overall, our findings demonstrate that the implementation of up to 25 preference signals has achieved multiple goals, including providing a metric for program directors to identify students interested in their program and reducing the total number of applications received. It is apparent that applicants face diminishing returns when applying to more than 25 residency programs, as the chances of receiving an interview are generally low. We expect that in future application cycles, the average number of applications submitted per applicant will near 25 as the low interview yield of additional applications is better understood.

This study is not without limitations. First, to encourage responses, we did not include any identifying information in our survey. While this allowed us to achieve a modest response rate of 35%, we are unable to fully assess whether the breadth of Oto‐HNS residency programs is fully represented in this study. As discussed above, it is likely our results are biased toward programs which receive a high volume of applicants and signals. Future studies investigating the relationship between interview invitations and preference signals among programs receiving a low total volume of signals are needed. Second, given all data was collected through survey, we are unable to confirm the accuracy of reported data. Nonetheless, interview trends since the implementation of high‐volume preference signaling is important to characterize in order to better advise future Oto‐HNS residents regarding the application process. These findings will continue to be relevant as the 2025 match will also utilize a 25 preference signal structure.

## Conclusions

In this study, we found that the implementation of 25 preference signals in the 2024 Oto‐HNS match has reduced the number of applications received by residency programs. For future Oto‐HNS residency applicants, our data suggests that applying to greater than 25 programs may result in diminishing returns, as the percent of applicants invited for an interview who did not signal the program nears 0%, while the percent of applicants invited for an interview who did signal the program is approximately 37%. Further studies are needed to confirm that these changes are consistent in future years of the Oto‐HNS residency match.

## Author Contributions

**Radhika Duggal**, design, data acquisition and analysis, manuscript writing and revision; **Kyra Osborne**, design, manuscript writing and revision; **Alan Kominsky**, design, manuscript writing and revision; **William S. Tierney**, design, data acquisition and analysis, manuscript writing and revision.

## Disclosures

### Competing interests

We have no conflicts of interest to disclose.

### Funding source

None.
